# The Engineered Thymidylate Kinase (TMPK)/AZT Enzyme-Prodrug Axis Offers Efficient Bystander Cell Killing for Suicide Gene Therapy of Cancer 

**DOI:** 10.1371/journal.pone.0078711

**Published:** 2013-10-23

**Authors:** Takeya Sato, Anton Neschadim, Arnon Lavie, Teruyuki Yanagisawa, Jeffrey A. Medin

**Affiliations:** 1 Molecular Pharmacology, Tohoku University, Sendai, Miyagi, Japan; 2 Research & Development, Canadian Blood Services, Toronto, Ontario, Canada; 3 Biochemistry and Molecular Genetics, University of Illinois at Chicago, Chicago, Illinois, United States of America; 4 Department of Medical Biophysics, University of Toronto, Toronto, Ontario, Canada; 5 Institute of Medical Science, University of Toronto, Toronto, Canada; 6 University Health Network, Toronto, Ontario, Canada; University of Pécs Medical School, Hungary

## Abstract

We previously described a novel suicide (or ‘cell fate control’) gene therapy enzyme/prodrug system based on an engineered variant of human thymidylate kinase (TMPK) that potentiates azidothymidine (AZT) activation. Delivery of a suicide gene sequence into tumors by lentiviral transduction embodies a cancer gene therapy that could employ bystander cell killing as a mechanism driving significant tumor regression *in vivo*. Here we present evidence of a significant bystander cell killing *in vitro* and *in vivo* mediated by the TMPK/AZT suicide gene axis that is reliant on the formation of functional gap-junctional intercellular communications (GJICs). Potentiation of AZT activation by the engineered TMPK expressed in the human prostate cancer cell line, PC-3, resulted in effective bystander killing of PC-3 cells lacking TMPK expression – an effect that could be blocked by the GJIC inhibitor, carbenoxolone. Although GJICs are mainly formed by connexins, a new family of GJIC molecules designated pannexins has been recently identified. PC-3 cells expressed both connexin43 (Cx43) and Pannexin1 (Panx1), but Panx1 expression predominated at the plasma membrane, whereas Cx43 expression was primarily localized to the cytosol. The contribution of bystander effects to the reduction of solid tumor xenografts established by the PC-3 cell line was evaluated in an animal model. We demonstrate the contribution of bystander cell killing to tumor regression in a xenograft model relying on the delivery of expression of the TMPK suicide gene into tumors via direct intratumoral injection of recombinant therapeutic lentivirus. Taken together, our data underscore that the TMPK/AZT enzyme-prodrug axis can be effectively utilized in suicide gene therapy of solid tumors, wherein significant tumor regression can be achieved via bystander effects mediated by GJICs.

## Introduction

Gene therapy approaches utilizing recombinant oncoretroviral or lentiviral vectors to deliver genes that potentiate various pharmacologic therapies directly into solid tumors hold promise in treating solid malignancies that are difficult to remove surgically, such as cancers afflicting the brain [1]. Suicide gene therapy of cancer (SGTC), also termed gene-directed enzyme-prodrug therapy (GDEPT), typically relies on the intratumoral delivery of suicide genes that facilitate selective and localized activation of specific prodrugs into their cytotoxic effector derivatives. Transcriptional- and pseudotype-based targeting of virions to cancer cells further extend the potential applications to include metastatic lesions and expand the delivery method to systemic administration [2,3]. The significant challenges associated with delivering the suicide gene into each and every malignant cell are often overcome by relying on bystander cell killing, or bystander effects, which create a localized kill zone around the successfully transduced tumor cells that functionally express the suicide gene, thus enhancing the overall SGTC efficacy [4].

Several suicide genes have been characterized and evaluated thus far with respect to their utility for SGTC. Among them, herpes simplex virus-derived thymidine kinase (HSV-tk) is one of the most extensively studied suicide genes for SGTC [5]. HSV-tk and various catalytically-enhanced mutants of this enzyme have been widely used as a suicide gene in combination with the guanosine analogue, ganciclovir (GCV), for the treatment of various cancers [1,6]. HSV-tk converts the nontoxic GCV into GCV-monophosphate (GCV-MP), which is further phosphorylated by cellular kinases to produce the toxic metabolite, GCV-triphosphate (GCV-TP), which inhibits host-cell DNA replication resulting in the induction of apoptosis [7]. Bystander effects have been well-characterized in the HSV-tk/GCV suicide gene therapy system, and are thought to involve the diffusion of activated GCV, either mono- or multiply-phosphorylated forms, from HSV-tk-expressing cells to bystander cells through gap-junctional intercellular communications (GJICs) that connect the cytosols of adjacent cells in many solid tumors and permit the exchange of small molecular weight metabolites by diffusion [8]. A number of factors limit the overall efficacy of HSV-tk for use in SGTC including: poor activation of GCV by HSV-tk into its cytotoxic form, primarily associated with the rate-limiting step in GCV activation being phosphorylation of GCV-MP into GCV-DP by the cellular guanylate kinase (GMPK) [9]; limited cytotoxicity of GCV, in particular against slowly-growing tumors, given its limited mechanism of action that relies solely on DNA replication [10]; and, finally, the poor lipophilicity of GCV resulting in reduced bystander effects and a poor ability to cross the blood-brain barrier thus limiting applicability in brain-targeted SGTC [11]. Taken together, SGTC approaches utilizing the HSV-tk/GCV axis are thus constrained by limited cytotoxicity and issues with effective dosing with GCV, which may have to be used at concentrations that are systemically myelosuppressive to achieve significant tumor ablation.

We have previously described alternative enzyme prodrug systems for suicide (or ‘cell fate control’) gene therapy [12,13], one of which utilizes a catalytically-enhanced variant of the human thymidylate kinase (TMPK-F105Y) that has been enabled to potentiate the rapid activation of the prodrug azidothymidine (AZT) [12]. Catalytically, wild-type TMPK is relatively slow at the phosphorylation of AZT monophosphate, which is the bottleneck step in its activation pathway in mammalian cells. The F105Y mutation, adopted based on comparison of a homologous region of the yeast TMPK sequence, results in a variant enzyme that is much more robust at phosphorylating AZT monophosphate and less active on its natural substrate, thymidylate monophosphate [14]. This novel suicide gene therapy axis utilizes a human-origin enzyme with minimal potential to cause adverse immune responses directed against the transgene, as is observed with HSV-tk-based approaches. It also utilizes an enzyme that is catalytically robust and acts at the rate-limiting step of AZT activation. Further, it utilizes a prodrug whose cytotoxic form can efficiently target both dividing and non-dividing cells owing to two distinct cytotoxicity mechanisms [12]. Finally, it utilizes a prodrug that has better lipophilicity profile and is likely more suitable for use in SGTC. Specifically, AZT is estimated to be at least 30-times more lipophilic than GCV [11], which predicts better passive diffusion and increased suitability of the AZT prodrug for suicide gene therapy of solid tumors, as well as better delivery of the prodrug to the brain, for example. Owing to these superior characteristics of the TMPK/AZT suicide gene therapy axis, we set out to evaluate the suitability of this system and the magnitude of the bystander effects it would engender in SGTC.

In this study, we report that sustained expression of the catalytically enhanced, AZT-active TMPK variant, TMPK-F105Y, in the human prostate carcinoma cancer cell line, PC-3, by transduction with lentiviral constructs readily sensitizes these cells to treatment with AZT and facilitates enhanced cell killing via bystander effects both *in vitro* and *in vivo*. We demonstrate that the bystander killing effect, observed following AZT treatment, is largely dependent on the existence of functional GJICs and is abolished by treatment with a gap junction inhibitor. Furthermore, we show the Pannexin1, and not Connexin43, likely forms the functional gap junctions between PC-3 cells. These results highlight the utility of lentivirus-mediated SGTC based on the modified-TMPK/AZT system and warrant its further evaluation *in vivo* in specific models of cancer.

## Materials and Methods

### Ethics Statement

 Animal experimental procedures followed a protocol approved by the Animal Care Committee of the University Health Network (Toronto, ON, Canada). Animals were maintained at the Animal Resource Centre at the Princess Margaret Hospital (Toronto, ON, Canada).

### Cell culture

 Human prostatic adenocarcinoma PC-3 cell line was obtained from American Type Culture Collection (ATCC; Manassas, VA, USA) and maintained in RPMI (Roswell Park Memorial Institute)-1640 medium (Wako Pure Chemical Industries, Ltd., Osaka, Japan) supplemented with 10% fetal bovine serum (FBS; PAA Laboratories GmbH, Pasching, Austria), 100 units/mL of penicillin (Nakalai Tesque, Inc., Kyoto, Japan), 100 µg/mL of streptomycin (Nakalai Tesque, Inc.), and 250 ng/mL of Amphotericin B (Nakalai Tesque, Inc.) in a 37°C, 5% CO_2_ atmosphere at constant humidity. The human embryonic kidney-derived 293T cell line was obtained from American Type Culture Collection (ATCC; Manassas, VA, USA) and maintained in Dulbecco’s Modified Eagle Medium (D-MEM; Wako Pure Chemical Industries, Ltd.) supplemented with 10% FBS, 100 units/mL of penicillin, 100 µg/mL of streptomycin, and 2 mM L-glutamine (Wako Pure Chemical Industries, Ltd.). 

### Production of recombinant LV/TMPK and transduction of PC-3 cells

 The procedure for the production of vesicular stomatitis virus-glycoprotein (VSV-g)-pseudotyped recombinant lentivirus, reported previously by our group [12], was used with minor modifications. Briefly, transfection of three plasmids (pHR’ gene-transfer plasmid, the packaging plasmid pCMVR8.91, and the vesicular stomatitis virus glycoprotein envelope-encoding plasmid pMD.G) into 293T cells was conducted using a polyethyleneimine(PEI)-procedure [15] and the virus supernatants were collected at 48 hours post-transfection, filtered using a 0.45 µm filter, and concentrated at 50,000×g for 2 hours. PC-3 cells were infected with the concentrated virus stocks in the presence of 8 µg/ml of protamine sulfate. Infected cells were then single-cell cloned by limiting dilution. Transgene expression in the transduced PC-3 cells was confirmed by Western blot analysis using rabbit anti-human TMPK (kindly provided by Dr. Manfred Konrad, Max Plank Institute for Biophysical Chemistry, Göttingen, Germany). For this analysis, total cell lysates were resolved by 12.5% sodium dodecyl sulfate polyacrylamide gel electrophoresis (SDS-PAGE) and blotted onto a polyvinylidene difluoride (PVDF) filter membrane (Millipore, Billerica, MA). The membrane was blocked with 0.5% fat-free skim milk in 20 mM Tris-buffered saline with 0.05% Tween-20, pH 7.4 (TBS-T, Wako Pure Chemical Industries, Ltd.). The membrane was probed with the rabbit anti-human TMPK (diluted 1:5,000), and then with the appropriate horseradish peroxidase–conjugated secondary antibody (diluted 1:5,000, Pierce Biotechnology, Rockford, IL, USA). Equal protein loading was confirmed with a murine anti-GAPDH antibody (diluted 1: 5,000, Ambion. Austin, TX, USA). Membranes, following development with the Immobilon Western Chemiluminescent HRP substrate (Millipore, Billerica, MA, USA), were imaged using the LAS-1000 system (Fuji Film Corp., Tokyo, Japan) charged-couple device camera. Expression of the enhanced green fluorescent protein (eGFP) in the transduced single-cell clones was confirmed by flow cytometric detection (FACS Canto II, BD Biosciences, Franklin Lakes, NJ, USA). 

### Dye transfer assay using flow cytometry and confocal microscopic observation

 For calcein labeling, cells grown to semi-confluence were washed twice with PBS (without calcium and magnesium), and then stained for 15 min with 20 µM calcein-acetoxy methyl (calcein-AM; Doujin Chemical Co., Kumamoto, Japan) dissolved in PBS (without calcium and magnesium). Following staining with calcein, the cells were washed with RPMI-1640 containing 10% FBS. For PKH26-labelling, cells were washed in PBS and resuspended in Diluent C (Sigma-Aldrich, St. Louis, MO, USA) to a density of 10^7^ cells/mL and stained with 20 µM PKH26 (Sigma-Aldrich) dissolved in Diluent C for 5 min. Cells were then washed 3 times with RPMI-1640 containing 10% FBS. Both the calcein-labelled and PKH26-labelled cells were mixed and co-cultured in 6-well plates (Corning Incorporated, Corning, NY, USA) for 3 days. Dye transfer was analyzed by flow cytometry (FACS Canto II, BD Biosciences) by measuring the fluorescence of calcein detected at 514nm and PKH26 detected at 567nm. In a separate assay, 100 µM carbenoxolone (CBX; Sigma-Aldrich) was added to the cell cultures to examine its ability to block dye transfer. Further, to detect the molecules participating in the gap junction formation, immunostaining of PC-3 cells was performed. Briefly, PC-3 cells, adhered to Lab-TekII chamber slide (Nalge Nunc International Corp., Naperville, IL, USA), were fixed with 4% (w/v) buffered formalin (Wako Pure Chemical Industries, Ltd.) in 0.1 M phosphate buffer, pH 7.4, and permeabilized with 0.1% (v/v) TritonX-100 for 15 min. Cells were then blocked with 5% normal goat serum, and were sequentially reacted with primary antibody solution (1:100 dilution in PBS containing 1% bovine serum albumin (BSA)) at 4°C overnight, followed by incubation in PBS containing the secondary antibody (1:500 dilution in PBS containing 1% BSA) labeled with Alexa488 (Molecular Probes, Inc., Eugene, OR, USA) and counterstained with rhodamine phalloidin (Cytskelton, Inc., Denver, CO, USA) for 3hr at room temperature. Antibodies used in this study were as follows: rabbit anti-connexin43 antibody (Signalway Antibody, College Park, MA, USA), rabbit anti-pannexin1 antibody (EMD Millipore Corp., Billerica, MA, USA), and Alexa488-labeled goat anti-rabbit IgG antibody (Molecular Probes, Inc.). Fluorescence signals were analyzed using a confocal laser-scanning microscope LSM-5 and LSM System version 3.98 (Carl Zeiss, Oberkochen, Germany) at the Biomedical Research Core of the Tohoku University Graduate School of Medicine.

### Cell proliferation assays

 Cells were seeded in 96-well plates (Corning Incorporated) at 5 × 10^5^ cells/well in 200 μl of RPMI-1640 medium, supplemented as described above, and incubated with increasing concentrations of AZT (0, 0.1, 1, 10, 100 μM, and 1 mM; Sigma-Aldrich). The medium was refreshed daily. After 4 days of culture, cell viability was determined using the Cell Counting Kit-8 (Doujin Chemical Co.). For determination of the optimal cell ratio for subsequent bystander experiments, both eGFP-expressing cells and TMPK-F105Y-transduced cells were seeded onto 96-well plates at different ratios, and cultured in the presence of 10 µM AZT for 5 days. Cell viability was determined as above using the Cell Counting Kit-8 (Doujin Chemical Co.).

### Evaluation of apoptosis

 Cells were seeded in 6-well plates (Corning Incorporated) at 10^6^ cells/well in 5 ml of cell culture medium (RPMI-1640 supplemented with 10% FBS, antibiotics, and antimycotics, as described above), with or without 10 µM AZT. After 4 days of culture, annexin V staining was performed according to the manufacturer's protocol (Annexin V-APC; BD Pharmingen, San Diego, CA). To confirm the requirement of GJICs for bystander killing induced by AZT, 100 µM carbenoxolone (CBX; Sigma-Aldrich) was added simultaneously with AZT to the culture. Relative apoptotic indices were calculated as normalized ratios of apoptosis in AZT-treated to AZT-untreated cells. To examine the contribution of soluble factors secreted from AZT-treated cells, wild-type PC-3 cells were cultured for 5 days in the conditioned media collected from wild-type TMPK or TMPK-F105Y-expressing cells that were treated with 10 µM AZT for 5 days, followed by evaluation of apoptosis induction in these cells as above.

### Reactive oxygen species (ROS) assay

 Generation of intracellular reactive oxygen species (ROS) was monitored by the dihydroethidium (DHE) procedure [16]. Briefly, after the indicated treatment timepoints, cells were further incubated with DHE (Wako Pure Chemical Industries, Ltd.) for 30 min at 37°C in the dark. Fluorescence emission intensity at 525 nm (following excitation at 488 nm) was measured by a flow cytometer (FACS Canto II, BD Biosciences). The change in mean fluorescent intensity (MFI) of samples measured from each treatment group was expressed as a percentage of DHE fluorescence of untreated control cells.

### Evaluation of bystander effect in vivo following an intratumoral injection of LV/TMPK

Non-obese diabetic/severe combined immunodeficiency (NOD/SCID) mice (5–8 weeks old, purchased from Jackson Laboratories, Bar Harbor, ME) were maintained at the Animal Resource Centre at the Princess Margaret Hospital (Toronto, ON, Canada). Animals were injected on day 0 into their right dorsal flank with 4x10^6^ LV/eGFP-transduced PC-3 cells, suspended in 200 µl of phosphate-buffered saline (PBS). Approximately 10 µl of a LV/TMPK-F105Y (1.5x10^8^ IU/ mL) preparation was injected intratumorally on day 11. Animals in drug-treated groups received a dose of 50 mg/kg/day of AZT intraperitoneally for 6 days starting at day 12. The experimental groups were as follows: PC-3-TMPK-F105Y treated with AZT and PC-3-TMPK-F105Y treated with vehicle (PBS). Animals were euthanized at day 18; tumors were then harvested and weighed.

### Statistics

Statistical significance between groups was evaluated by one-way ANOVA analyses with the Bonferroni post-hoc test using GraphPad InStat (ver. 3.0a for Macintosh; GraphPad Software, San Diego CA).

## Results

### PC-3 cells express functional GJICs comprised of pannexin1

**Figure 1:**
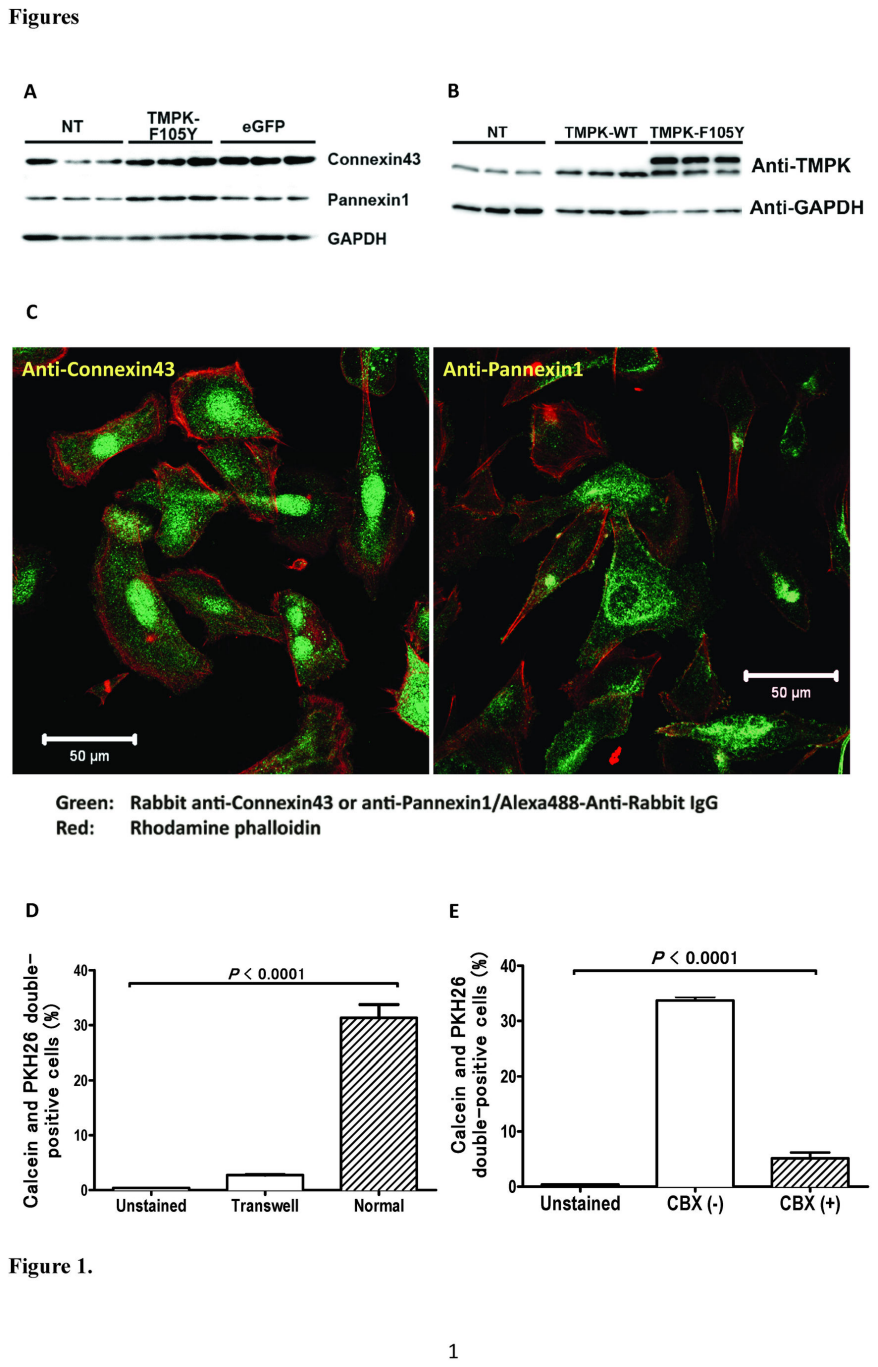
PC-3 cells express functional GJICs. (A) Expression of GJIC components, connexin43 and pannexin1, was confirmed by Western blot on whole-cell lysates of non-transduced, LV/TMPK-transduced, and LV/eGFP-transduced PC-3 cells. GAPDH expression was evaluated as an equal loading control. (B) Expression of TMPK was confirmed by Western blot on whole-cell lysates of non-transduced, LV/TMPK-transduced, and LV/TMPK-F105Y-transduced PC-3 cells. GAPDH expression was evaluated as an equal loading control. (C) Expression pattern of connexin43 (left panel) and pannexin1 (right panel) GJIC components (green fluorescence) was assessed by confocal immunofluorescent microscopy. Cytoskeleton (F-actin) was visualized with rhodamine phalloidin (red fluorescence) staining. (D) Dye transfer of calcein into adjacent PKH26-labelled cells was measured by flow cytometry in direct (normal) or transwell co-cultures of PC-3 cells as percentage of cells double-positive for calcein and PKH26 fluorescence (n=3). (E) Dye transfer of calcein into adjacent PKH26-labelled cells was significantly inhibited by carbenoxolone (CBX) (n=3). Statistical significance is indicated (p<0.0001).

Gap junctional intracellular communications (GJICs), in addition to playing a direct role in tumor progression [17], are considered to be important for the bystander cell killing effect in SGTC applications [18]. GJICs are typically comprised of proteins of the connexin family, and, as was recently shown, from proteins of the pannexin family as well [19]. GJICs allow for the passive diffusion of small molecules (≤1 kDa in size) between adjacent, connected cells. The expressions of key GJIC proteins, connexin43 and pannexin1, were confirmed in PC-3 cells by Western blot analysis (Figure 1A); expressions of wild-type TMPK and TMPK-F105Y were also confirmed analogously (Figure 1B). The distribution patterns of connexin43 and pannexin1, as determined by confocal immunofluorescent microscopy analysis, revealed however that PC-3 cells predominantly express connexin43 in cytoplasmic perinuclear compartments (Figure 1C, left panel), whereas pannexin1 was primarily localized to the plasma membrane, observed as spots and streaks that are the hallmark of the GJIC appearance pattern (Figure 1C, right panel). This finding suggests that pannexin1 may predominate over connexin43 in the formation of functional GJICs in this prostate cancer cell line. 

We next examined the formation of functional GJICs in PC-3 cells in a dye transfer experiment. Two cell populations, separately and stably labelled with one of two different dyes (calcein, which is non-membrane permeable and is trapped in the cell cytosol, and PKH26, which specifically labels cell membranes) exhibited dye transfer in 2-day long co-cultures, as demonstrated by the appearance of doubly-labelled cells (Figure 1D). This calcein dye transfer to PKH26-labelled cells required direct cell-cell contact, as the dye transfer was not apparent in differentially labelled cells that were cultured in a transwell system (Figure 1D). Further, dye transfer was significantly inhibited in the presence of a specific gap junction inhibitor, carbenoxolone (CBX) (Figure 1E). Taken collectively, the data suggests that calcein dye transfer into PKH26-labelled cells required direct cell-cell contact, and was likely facilitated by functional GJICs. 

### Evaluation of AZT-mediated cell killing of LV/TMPK-transduced PC-3 cells and bystander effects

**Figure 2:**
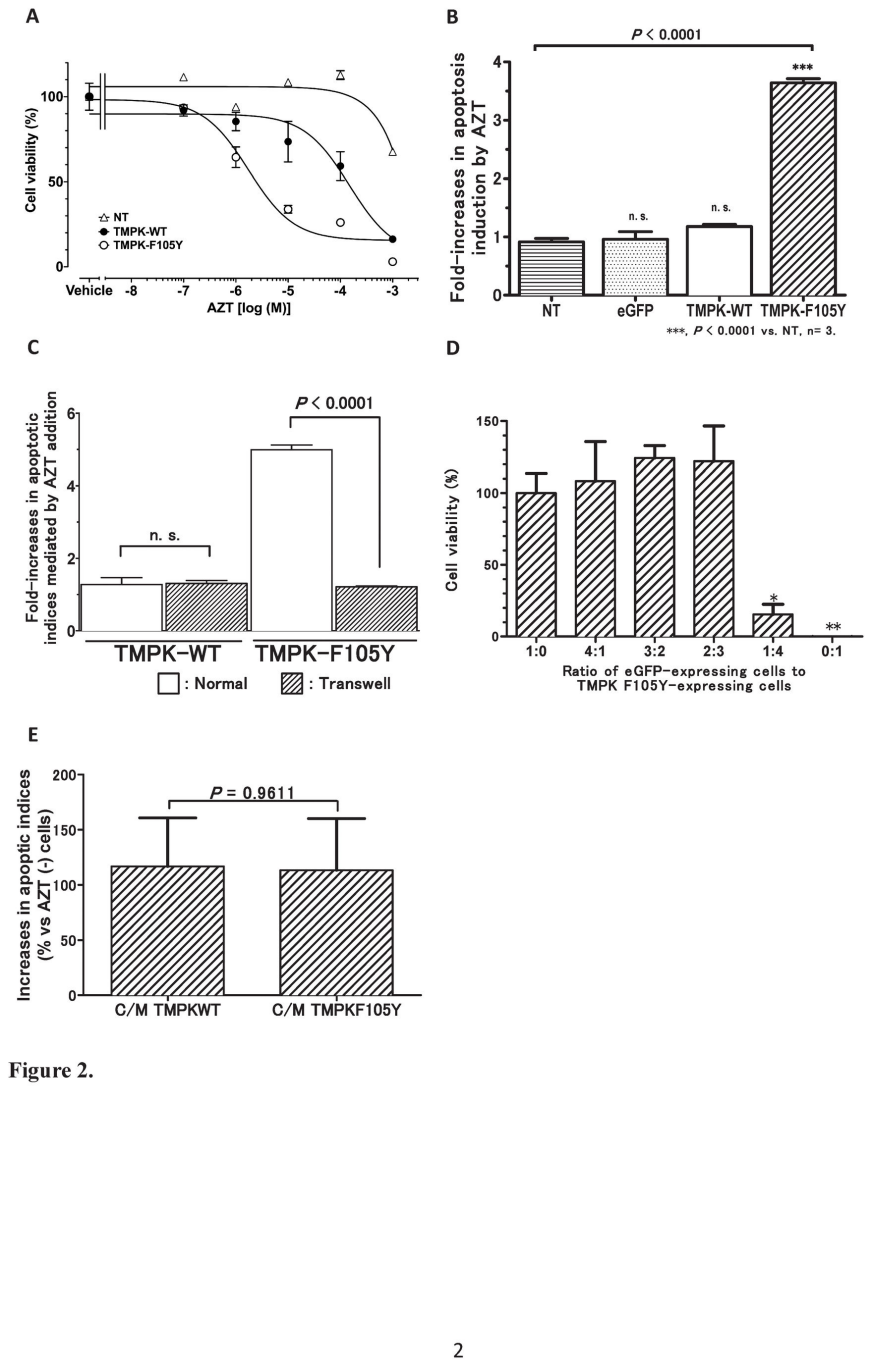
AZT activation by TMPK-F105Y results in bystander cell killing by a mechanism that requires direct cell-to-cell contact. (A) Dose-dependent cell killing of LV-transduced PC-3 cells incubated with increasing concentrations of AZT (mean +/- SEM, n=3). (B) Induction of apoptosis by 10 µM AZT was evaluated by annexin V staining of treated TMPK-F105Y cells. (C) Bystander cell killing was assessed by relative evaluation of annexin V staining in AZT-treated to untreated bystander eGFP-expressing PC-3 cells by flow cytometry in direct (normal) and transwell co-cultures of eGFP-transduced with TMPK-WT- or TMPK-F105-transduced PC-3 cells (n=3). Statistical significance is indicated (p<0.0001). (D) Determination of the optimal ratio of eGFP-expressing cells to TMPKF105Y-expressing cells to evaluate bystander killing effects by 10 µM AZT (mean +/- SEM, n=4; * p < 0.05, ** p < 0.01). (E) Induction of apoptosis in wild-type PC-3 cells by conditioned media from AZT-treated wild-type TMPK-transduced and TMPK-F105Y-transduced PC-3 cells normalized to apoptosis in cells treated with conditioned media from AZT-untreated cells (n=3).

PC-3 cells were transduced with lentiviral vectors engineering expression of either wild-type TMPK or TMPK-F105Y. TMPK and TMPK-F105Y expression was confirmed by Western blot analysis using a rabbit anti-human TMPK antibody in individual cell clones isolated by limiting dilution (Figure 1B). While endogenous TMPK expression was detectable in the non-transduced (NT) cells, a significant overexpression of TMPK was observed in the transduced cells. Dose-dependent AZT sensitivity of PC-3 cell clones expressing wild-type TMPK, which are unable to activate AZT significantly, or the AZT-active TMPK-F105Y mutant, was assessed in cell culture. PC-cells expressing the TMPK-F105Y mutant were highly sensitive to treatment with increasing concentrations of AZT (IC50 of 1.8 μM) compared to non-transduced cells or cells overexpressing wild-type TMPK (IC50 of >1 mM and >0.1 mM, respectively) (Figure 2A). Based on the determined dose-responses, a dose of 10 μM of AZT was selected for subsequent experiments as a dose that was very effective in TMPK-F105Y-expressing cells but exhibited no detectable toxicity in non-transduced cells or cells overexpressing wild-type TMPK. 

To further confirm the specific induction of apoptosis in TMPK-F105Y-expressing cells following incubation with 10 µM AZT, we assessed the exposure of an apoptotic marker, phosphatidylserine (PS), on the cell surface of treated cells by staining with APC-conjugated annexin V protein. TMPK-F105Y-transduced cells cultured in the presence of 10 µM AZT, but not other control cell groups, exhibited a significant induction of apoptosis as shown by a more than 3-fold increase in the apoptotic index of these cells (Figure 2B).

To evaluate the bystander cell killing mediated by TMPK-F105Y-derived activation of AZT in PC-3 cells, TMPK-transduced and non-transduced control cells were directly co-cultured with independent PC-3 cells engineered to stably express the enhanced green fluorescent protein (eGFP). Co-cultures treated with AZT were evaluated by annexin V (and 7-AAD) staining for induction of apoptosis in the eGFP-positive PC-3 cell population. As shown in Figure 2C (white bars), only eGFP-positive bystander cells co-cultured with cells expressing the AZT-active TMPK-F105Y and not the wild-type TMPK demonstrated a significant increase in the apoptotic index upon AZT treatment. TMPK-expressing effector cells and eGFP-expressing bystander cells needed direct cell-cell contact, since no increase in the apoptotic index of the bystander cells was observed when the cell populations where separated by a transwell system (Figure 2C, shaded bars). The bystander killing effect in co-cultures with a 1:4 ratio of eGFP-expressing cells (20%) to TMPK-F105Y-expressing cells (80%) was highly significant leading to an overall 80% decrease in cell survival in the co-cultures following 5 days of AZT treatment (Figure 2D), suggesting that a potent bystander killing effect could be observed. To further confirm that the bystander effect observed was not mediated by soluble factors secreted from TMPK-F105Y-expressing, AZT-treated cells, we cultured non-transduced PC-3 cells in the conditioned media collected from wild-type TMPK or TMPK-F105Y-expressing cells treated with 10 µM AZT for 5 days. Under these culture conditions, non-transduced PC-3 cells did not exhibit significant cell death (Figure 2E). Taken together, these data indicate that the bystander killing effect observed is mediated by the transfer of cytotoxic AZT metabolites by a mechanism that requires direct cell-to-cell contact.

### The role of GJICs in mediating the bystander effect in PC-3 cells

**Figure 3:**
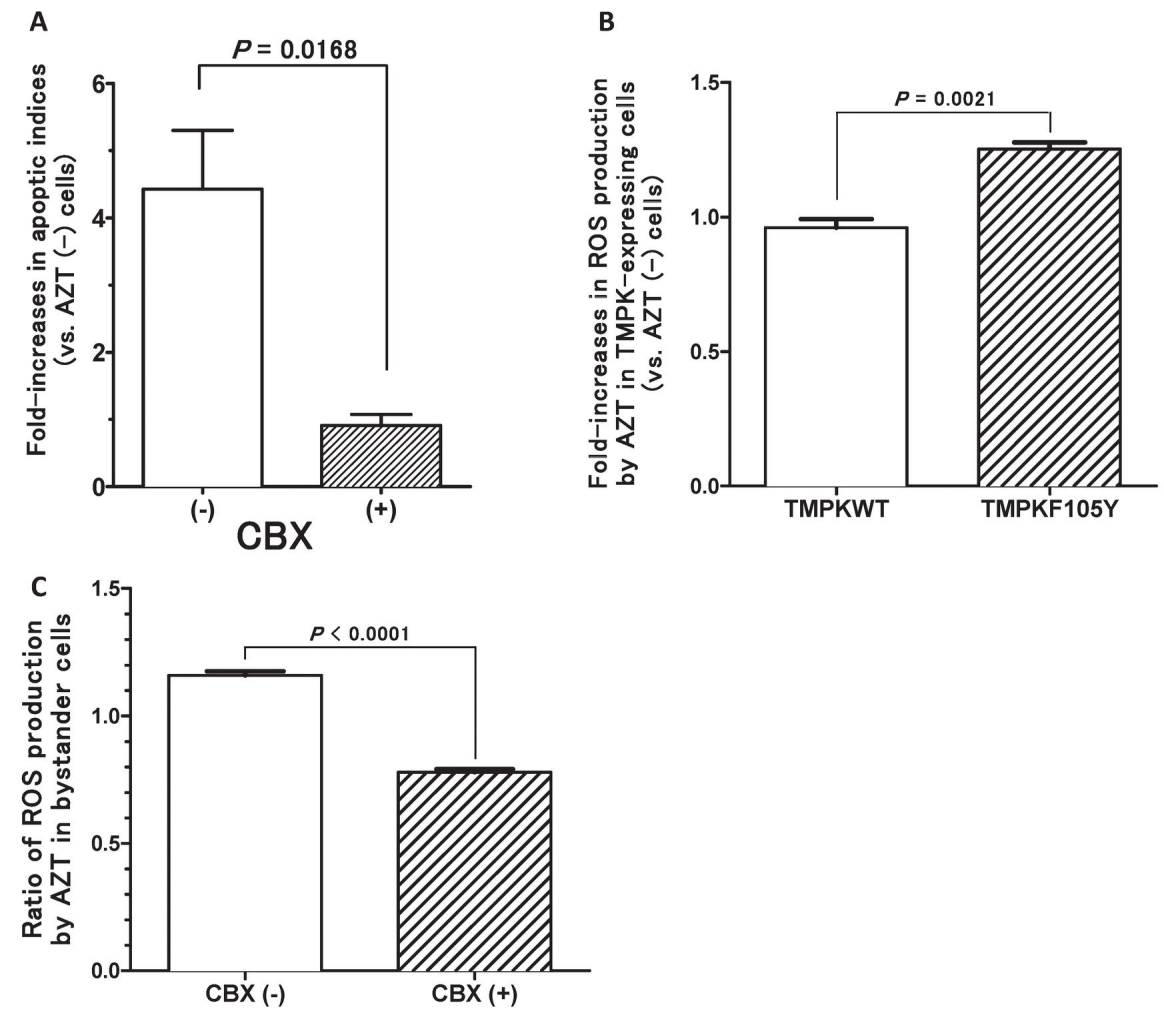
AZT-mediated bystander cell killing is dependent on GJICs and is inhibited by carbenoxolone. (A) Bystander cell killing was assessed by relative evaluation of annexin V staining in AZT-treated bystander eGFP-expressing PC-3 cells, co-cultured with TMPK-F105Y-tranduced PC-3 cells, by flow cytometry. Co-cultures were either left untreated or treated with the pan GJIC inhibitor, carbenoxolone (CBX) (n=3). (B) Fold-change in the production of reactive oxygen species (ROS) due to AZT treatment was evaluated in wild-type TMPK-expressing and TMPK-F105Y-expressing PC-3 cells (n=3). (C) Fold change in the production of reactive oxygen species (ROS) due to AZT treatment was evaluated in eGFP-expressing PC-3 cells, co-cultured with TMPK-F105Y-tranduced PC-3 cells, with and without carbenoxolone (CBX) treatment (n=3). Statistical significance is indicated (p<0.0001).

To further explore the mechanisms of the bystander effect observed in the PC-3 cells facilitated by the TMPK-F105Y/AZT suicide system, we assessed the bystander cell killing observed in the eGFP-positive population directly co-cultured with TMPK-F105Y-expressing cells treated with 10 µM AZT in the absence and presence of 100 µM of a specific gap junction inhibitor, carbenoxolone (CBX). Addition of CBX to the co-culture completely abolished bystander cell killing (apoptotic index of 1) (Figure 3A), indicating that the bystander effect in our PC-3 co-cultures is mainly mediated by functional GJICs. 

We determined that AZT activation in TMPK-F105Y-expressing cells increases reactive oxygen species (ROS) production (Figure 3B). Since it is well known that ROS production often induces cellular damage leading to cell death, we speculated that production of ROS could contribute to the bystander cell killing observed in this system. Indeed, we have shown that CBX treatment of our co-cultures that would disrupt functional GJICs resulted in the reduction of ROS produced following AZT treatment in the bystander cell population (Figure 3C), suggesting that the transfer of activated AZT metabolites to bystander cells induces the production of ROS and potentially contributes to the induction of cellular apoptosis in these cells.

### Evaluation of AZT-mediated bystander effects in human prostate cancer xenograft models in vivo

**Figure 4:**
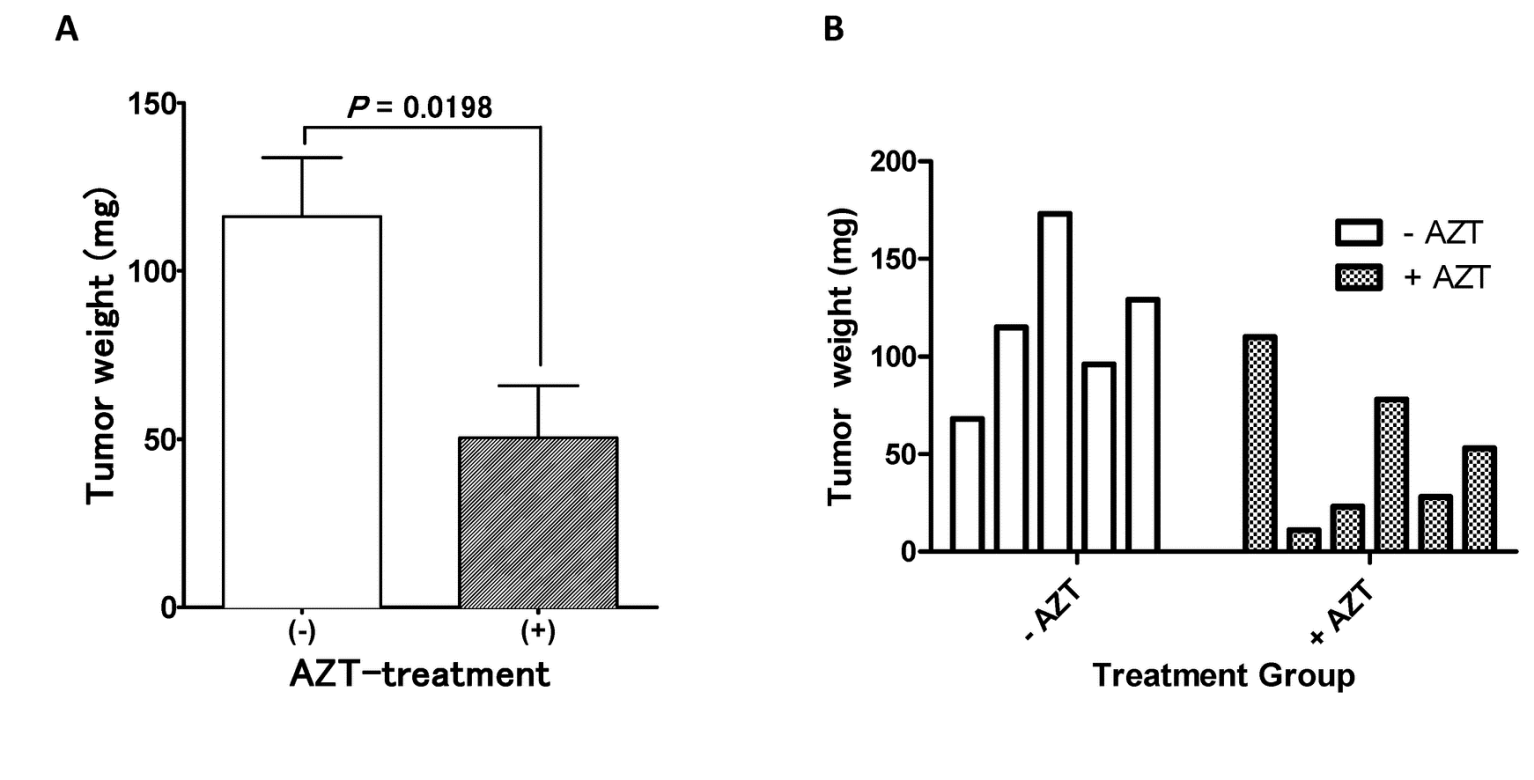
Bystander cell killing mediated by TMPK-F105Y/AZT therapy drives a significant tumor mass reduction in a prostate cancer xenograft model. (A) Magnitude of bystander cell killing was evaluated following the delivery of the TMPK-F105Y suicide gene by direct intratumoral injection of LV/TMPK into established tumors in NOD/SCID mice (n=6). Reduction in tumor mass was assessed at the end of 6-day AZT treatment regimen (at 50mg/kg/day) by extraction of tumors and measurement of wet tumor weight. Statistical significance is indicated by an asterisk (* p<0.05). (B) Weight of individual extracted tumors is shown for each animal in the AZT-treated and vehicle-untreated groups.

To evaluate the efficacy of bystander effects induced in the TMPK-F105Y/AZT suicide system *in vivo* and the suitability of this approach for SGTC, we employed a PC-3 xenograft mouse model. Since poor transduction of tumor cells by vectors engineering the expression of suicide genes is a general limitation of GDEPT approaches, we sought to examine whether bystander effects in the TMPK/AZT suicide system are sufficiently robust to possibly compensate for limitations in tumor transduction efficiencies. Here NOD/SCID animals were subcutaneously inoculated with non-transduced PC-3 tumors, and after 11 days of tumor growth, the palpable tumors were directly injected, intratumorally, with ~1.5x10^6^ infectious viral particles engineering the expression of the TMPK-F105Y suicide gene. Starting 24 hours following the injection of the virions, the animals received daily intraperitoneal injections of AZT (50 mg/kg/day) or a vehicle control for 6 consecutive days. Mice were euthanized at the end of the treatment period, and tumor tissues were harvested and weighed. Tumors extracted from AZT-treated animals injected with the TMPK-F105Y-engineered lentivirus were significantly reduced compared to tumors from untreated animals (Figure 4A), and several tumors in the AZT-treated animals showed substantial growth impairment (Figure 4B). To put this result in context, we had previously assessed our ability to transduce PC-3 tumors with eGFP-engineering lentivirus by direct intratumoral injection and achieved transduction rates of up to 20% at best with a marbled expression pattern, mainly localized to the lentivirus injection sites [20], suggesting that the more than 50% reduction in tumor weight observed here in the AZT-treated tumors was mediated by localized bystander cell killing in this TMPK/AZT suicide gene therapy system.

## Discussion

A crucial limitation of suicide gene therapy approaches directed at the treatment of cancer is the inability to transduce each and every malignant cell - with typical transduction efficiencies being <1% in the clinic with non-replicating viral vectors (for example, typical vector integration observed in a recent study is less than 500 copies per μg of DNA – an equivalent of 0.01 copies per cell [1]). This limitation, however, can be overcome with reliance on localized bystander effects that further augment and compound the therapeutic potential of enzyme/prodrug-based suicide gene therapies. Thus, understanding and improving bystander cell killing is fundamental to improving the clinical success of SGTC. 

We have previously demonstrated GJIC-dependent bystander effects with suicide gene therapy based on the delivery of a catalytically-enhanced variant of the deoxycytidine kinase (dCK) suicide gene in a U87 model of glioblastoma-astrocytoma [21]. In this present work we employed another one of our novel suicide gene therapy systems, which is based on an engineered variant of human TMPK that activates AZT, against cancer. We investigated the *in vitro* and *in vivo* efficacy and mechanisms of secondary bystander cell killing effects. Bystander effects can be mediated by the free passive diffusion of toxic antimetabolites through cell membranes [11] or may depend on gap junctional intercellular communications - membrane structures forming intercellular channels that allow the diffusion of small hydrophilic molecules (typically under 1 kDa in size) through the cell membranes of adjacent cells [22,23]. The former mechanism has apparent advantages for cancer-directed gene therapy, since many tumor cells have been reported to lack functional GJICs [24]. However, this approach may suffer from the risk of systemic diffusion of toxic metabolites generated within the tumor, potentially resulting in significant off-target toxicity. On the other hand, bystander effects mediated by cytotoxic metabolites diffusing through GJICs in tumors that are interconnected by these communication channels are more likely to be confined to the tumor tissue alone, which would be true for both primary tumors and metastases. 

Given the inherent limitations of the HSV-tk-based suicide gene therapy system, more efficient prodrug-activating systems are desirable for successful clinical application of SGTC that would permit the rapid diffusion and accumulation of cytotoxic metabolites in bystander cells. This is a particularly important requirement since many cancers develop multi-drug resistance by upregulating the expression of membrane efflux pumps [25], which are capable of rapidly removing cytotoxic prodrug metabolites from the cytoplasm. Finally, enzyme-prodrug systems that can target both dividing and non-diving cells may be required to achieve maximal tumor eradication in certain malignancies. The poor catalytic efficiency of the HSV-tk/ganciclovir-based suicide gene therapy system may have yielded mediocre bystander effects in previous work [26], examining bystander killing of the aggressive PC-3 prostate cancer cells. We hypothesize that the HSV-tk/ganciclovir system may not meet certain thresholds required for efficacious killing of bystander cells in PC-3 cells, and that our novel TMPK/AZT system [12] would result in better bystander effects in that same cancer model. We have therefore evaluated the magnitude of bystander effects mediated by our TMPK/AZT-based suicide gene therapy in PC-3 cells *in vitro* and *in vivo*. This unique system is based on an active-site engineered human TMPK, TMPK-F105Y, which is capable of selectively activating AZT. It is characterized by catalytic robustness, rapid antimetabolite accumulation as the enzyme overcomes the rate-limiting step in the AZT activation pathway, and cell killing driven by multiple mechanistic pathways, with toxicity against both DNA-replicating and non-dividing cells.

Despite other reports that PC-3 cells did not exhibit strong bystander effects with the HSV-tk/GCV system [26,27], we were able to observe fairly significant bystander cell killing both *in vitro* and *in vivo* with our TMPK-F105Y/AZT suicide gene therapy. Our findings indeed support the lack of proper intracellular localization of connexin43 expression in PC-3 cells, but clearly indicate the existence of functional GJICs in these cells by dye-transfer experiments, which we believe are comprised at least in part of the pannexin family of proteins [28,29]. Bystander cell killing effect was completely abolished with the physical separation of the effector and bystander cells in transwell experiments as well as upon GJIC inhibitor treatment, highlighting the requirement for functional GJICs and indicating that the bystander effects observed in the TMPK/AZT system are not mediated by any soluble factor or free diffusion of antimetabolites to bystander cells. 

Finally, we underscore the therapeutic utility of the TMPK/AZT system for SGTC in a robust *in vivo* model of human prostate cancer xenografts. This study, in which efficient tumor regression was observed with significant contribution from bystander effects, provides the preclinical proof-of-principle for the application of the TMPK/AZT suicide gene system for lentiviral-based GDEPT of solid malignancies.
